# Long-term disease-free survival following comprehensive involved site radiotherapy for oligometastases

**DOI:** 10.3389/fonc.2023.1267626

**Published:** 2023-12-05

**Authors:** Johnny Kao, Michelle Sahagian, Vani Gupta, Symeon Missios, Ashish Sangal

**Affiliations:** ^1^ Department of Radiation Oncology, Good Samaritan University Hospital, West Islip, West Islip, NY, United States; ^2^ New York Institute of Technology College of Osteopathic Medicine, Old Westbury, NY, United States; ^3^ Cancer Institute, Good Samaritan University Hospital, West Islip, NY, United States; ^4^ Long Island Brain and Spine, West Islip, NY, United States

**Keywords:** oligometastases, radiation therapy, minimal residual disease, palliative care, stereotactic body radiation therapy

## Abstract

**Introduction:**

Despite recent advances in drug development, durable complete remissions with systemic therapy alone for metastatic cancers remain infrequent. With the development of advanced radiation technologies capable of selectively sparing normal tissues, patients with oligometastases are often amenable to comprehensive involved site radiotherapy with curative intent. This study reports the long-term outcomes and patterns of failure for patients treated with total metastatic ablation often in combination with systemic therapy.

**Materials and methods:**

Consecutive adult patients with oligometastases from solid tumor malignancy treated by a single high volume radiation oncologist between 2014 and 2021 were retrospectively analyzed. Oligometastases were defined as 5 or fewer metastatic lesions where all sites of active disease are amenable to local treatment. Comprehensive involved site radiotherapy consisted of stereotactic radiotherapy to a median dose of 27 Gy in 3 fractions and intensity modulated radiation therapy to a median dose of 50 Gy in 15 fractions. This study analyzed overall survival, progression-free survival, patterns of failure and toxicity.

**Results:**

A total of 130 patients with 209 treated distant metastases were treated with a median follow-up of 36 months. The 4-year overall survival, progression-free survival, local control and distant control was 41%, 23%, 86% and 29%. Patterns of failure include 23% alive and free of disease (NED), 52% distant failure only, 9% NED but death from comorbid illness, 7% both local and distant failure, 4% NED but lost to follow-up, 4% referred to hospice before restaging, 1% local only failure, 1% alive with second primary cancer. Late grade 3+ toxicities occurred in 4% of patients, most commonly radionecrosis.

**Conclusion:**

Involved site radiotherapy to all areas of known disease can safely achieve durable complete remissions in patients with oligometastases treated in the real world setting. Distant failures account for the majority of treatment failures and isolated local failures are exceedingly uncommon. Oligometastases represents a promising setting to investigate novel therapeutics targeting minimal residual disease.

## 1 Introduction

There is great enthusiasm for advances in drug development targeting distant metastases from solid tumors in the mainstream media (1). Despite significant progress, metastatic cancer remains largely incurable and results in approximately 90% of cancer deaths (2, 3). Following treatment with either immunotherapy or molecularly targeted systemic therapies alone, responses are uncommon benefiting less than 13% of all cancer patients (4, 5). Published evidence dating to the late 2000’s established the long-term curative potential of radiation therapy to all areas of known disease for patients with oligometastases (6–8). Two randomized trials demonstrated improved progression-free survival and overall survival when comprehensive local consolidative therapy is added to systemic therapy alone for patients with oligometastases from non-small cell lung cancer or mixed primary tumors (9, 10). By contrast, adding stereotactic radiation to some but not all distant metastases fails to improve outcomes compared to immunotherapy alone (11–13).

In the real world setting, patients with less than 5 distant metastases represent approximately 30% of patients requiring radiation therapy for metastatic disease (14). While much of the published evidence of radiation therapy for extracranial oligometastases focused narrowly on stereotactic body radiation therapy for well selected patients, real world patients include clinical presentations requiring alternative modes of radiation therapy including stereotactic radiosurgery for brain metastases or intensity modulated radiation therapy for a bulky primary tumor and regional nodes (9, 15, 16). We hypothesized that the development of advanced radiation technologies capable of sparing normal tissues at risk along with appropriate risk stratification would allow for the safe and effective application of comprehensive involved site radiation for a broader group of patients with oligometastases seen in the context of a busy community hospital practice (17).

## 2 Materials and methods

### 2.1 Patient selection

This study was approved by the Good Samaritan University Hospital IRB #16-016 with waiver of informed consent. The study population included consecutive patients ≥18 years of age with pathologically confirmed solid tumor malignancy with oligometastases referred to a single high volume radiation oncologist. Oligometastases were defined as 5 or fewer active metastatic lesions on whole body imaging where all sites of active disease, including the primary tumor and involved regional lymph nodes, were amenable to treatment. For patients with metachronous metastases, the primary tumor was controlled with prior local therapy. Whole body imaging included PET/CT, CT chest, abdomen and pelvis, bone scan or MRI of the brain or spine as per National Comprehensive Cancer Network guidelines for specific primary tumors.

Relevant baseline patient characteristics include ECOG performance status, primary tumor and histology, pre-treatment serum albumin, ESTRO/EORTC oligometastatic disease classification, age, gender, metastasis site, number of metastases treated, cumulative GTV volume, radiation dose and number of fractions for each treatment site, whether the primary tumor was also treated and systemic therapy (18). Diverse radiation dose schedules to primary tumor and metastases were converted to a biological equivalent dose (BED) using the formula BED = D x [1+d/(α/β)] where D is total dose delivered, d is dose per fraction and α/β=10 for malignant tumors. This information was extracted from retrospective EPIC and Aria chart review.

### 2.2 Treatment and follow-up

Patient immobilization was highly personalized based on location. All patients underwent CT simulation and contouring and external beam radiotherapy treatment planning was performed on Eclipse. When appropriate, MRI, PET or CT with contrast was imported and fused to assist with accurate target delineation. Depending on location, volume and organs at risk, intensity modulated radiation therapy, stereotactic body radiotherapy or stereotactic radiosurgery was prescribed with PTV expansions as appropriate. The GTV (or ITV for tumors with organ motion) received ≥100% of the prescribed dose and the PTV received a minimum of ≥95% of the prescribed dose. When conflicting, organ at risk dose limits were prioritized over PTV coverage. Image-guided radiation therapy was delivered on the Varian TrueBeam or Varian Edge equipped with a 6-degree of freedom robotic couch and cone beam CT. Brachytherapy planning was performed on Oncentra and delivered using Nucleotron high dose rate brachytherapy. A small subset of patients underwent surgery (most commonly craniotomy) or interventional radiology ablation in addition to radiotherapy.

Systemic therapy was administered at the discretion of the treating medical oncology and/or urologist. Prior to radiation, 68% were not actively receiving systemic therapy while 32% received systemic therapy with diverse treatment regimens (**Supplementary Table 1**). During or following radiation, 74% received systemic therapy and 26% received no systemic therapy. Systemic treatment regimens included 18% chemotherapy alone, 15% hormonal therapy with or without androgen receptor inhibitor or CDK4/6 inhibitor, 12% immunotherapy alone, 10% chemotherapy combined with biologically targeted therapy, 9% biologically targeted therapy alone, 9% chemoimmunotherapy and 2% hormonal therapy with chemotherapy or targeted therapy.

During radiotherapy, patients were assessed weekly. Following radiotherapy, patients were followed by radiation oncology and medical oncology using EPIC and supplemented by tumor imaging and blood work. In the community hospital setting, follow-up is quite robust with scheduled outpatient follow-up supplemented by a daily inpatient huddle jointly attended by both medical oncology and radiation oncology.

### 2.3 Outcomes

The primary endpoints were overall survival and progression-free survival using the Kaplan Meier method measured from date of consultation until death or most recent follow-up. Potential predictors of survival were assessed using the log-rank test using cutpoints validated in the published literature. Variables with a p value of <0.10 were entered into Cox multivariable regression analysis. Treatment failures were further classified to estimate local control and distant control on a per patient basis. Patient and treatment characteristics were reported with median and interquartile ranges (IQR) for continuous variables. Acute and late toxicities were scored using the Common Terminology Criteria for Adverse Events (CTCAE) version 5.0. Statistical analysis was performed using Stata version 13.1.

## 3 Results

### 3.1 Patient and treatment characteristics

Between 1/2014 and 12/2021, a total of 130 patients with 209 targeted distant metastases were treated by a single radiation oncologist. Patient and disease characteristics were summarized in **Table 1**. The most common primary tumors were lung (35%), prostate (12%) and breast (9%). The median follow-up among surviving patients was 35.2 months (IQR 19.5 to 64.1 months).

**Table 1:** Characteristics of 130 patients with oligometastases.

Variable	Percent (number)	Median Overall Survival (months)	P value
Age, Median (range)	71 (28 to 96)		0.02
<70	45% (58)	66.0	
≥70	55% (72)	27.0	
Gender			0.26
Male	54% (70)	45.3	
Female	46% (60)	28.9	
ECOG performance status			0.003
0	23% (30)	Not reached	
1	48% (63)	28.9	
2	22% (28)	18.6	
3 or 4	7% (9)	12.8	
Category of oligometastatic disease			0.98
Synchronous oligometastases	40% (52)	42.8	
Metachronous oligorecurrence or oligoprogression	37% (48)	36.1	
Other (induced or repeat oligorecurrence oligoprogression oligopersistence)	23% (30)	31.7	
Primary tumor			
Lung	35% (45)	43.9	0.92
Prostate	12% (15)	67.9	0.03
Breast	9% (12)	82.9	0.56
Colorectal	8% (10)	42.8	0.88
Endometrial	8% (10)	31.7	0.56
Melanoma	5% (6)	24.6	0.08
Occult primary	5% (6)	7.1	0.07
Hepatobiliary and pancreatic	4% (5)	15.5	<0.01
Other *	16% (21)	33.4	0.41
Favorable primary tumor			0.02
Breast, prostate or kidney		67.9	
All others		28.9	
Metastasis location	209 tumors		
Bone	31% (40)	36.1	0.46
Brain	30% (39)	28.9	0.49
Lung	22% (28)	39.8	0.71
Distant Lymph Nodes	19% (25)	45.2	0.80
Liver	10% (13)	9.9	0.15
Adrenal	3% (4)	12.2	0.64
Albumin			<0.001
≥3.4	66% (86)	45.2	
<3.4	28% (33)	15.5	
Unknown	8% (11)	31.7	
Number of metastases treated			0.43
0	7% (9)	31.7	
1	56% (72)	36.1	
2 to 5	36% (47)	32.3	
Cumulative GTV in cm^3^, median (range)	44.1 (0.1 to 562.6)		0.25
<27.7 cc	39% (51)	43.9	
≥27.7 cc	61% (79)	31.7	
Primary tumor BED			0.51
<75 Gy	25% (33)	31.7	
≥75 Gy	22% (28)	54.1	
Primary tumor not treated	53% (69)	33.4	
Average metastasis BED			0.73
<75 Gy	61% (79)	36.1	
75 to 99.9 Gy	22% (28)	28.9	
≥100 Gy	18% (23)	42.8	
Adjvuant systemic therapy			0.32
Yes	74% (96)	36.1	
No	26% (34)	43.9	

*Other primary sites include ovary 3%, gastroesophageal 3%, non-melanoma skin 3%, renal 2%, sarcoma 2%, cervix 2%, thyroid 1%.

**Figure 1:**
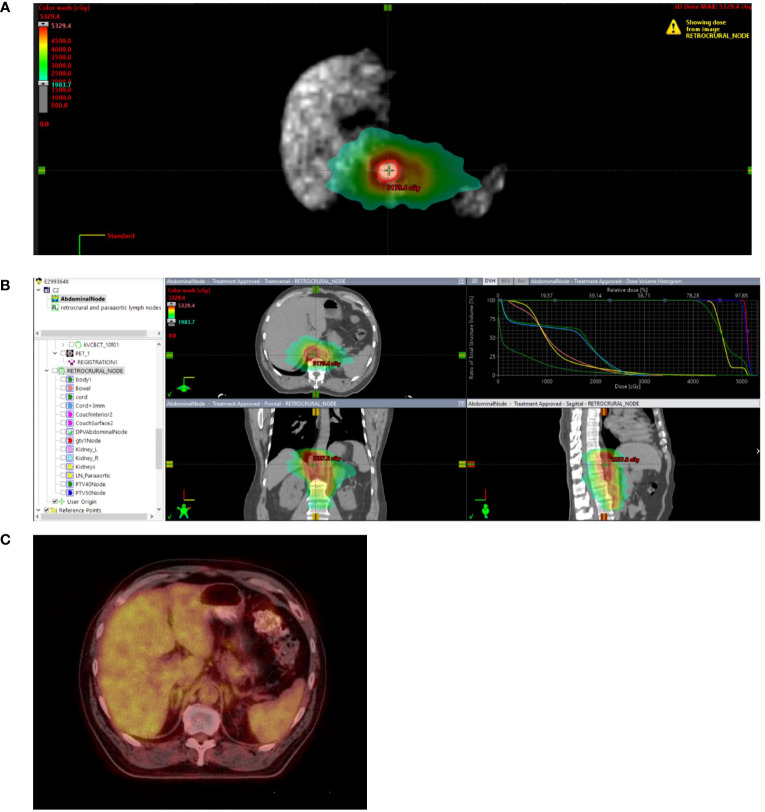
Stage IIIB rectal cancer initially treated with total neoadjuvant therapy followed by sphincter-sparing surgery with pathologic complete response followed by 2 additional cycles of adjuvant FOLFOX. While on surveillance, the patient presented with an elevated CEA of 18.3. PET/CT demonstrated a new 3.5 cm retrocrural node with a SUVmax of 3.5 without additional areas of FDG avid disease. Biopsy confirmed metastatic rectal adenocarcinoma. **(A)** Treated with involved site radiotherapy to 50 Gy in 10 fractions to the PET positive node while covering PET negative prominent paraaortic nodes to 40 Gy in 10 fractions. **(B)** Radiation plan demonstrating selective sparing of uninvolved bowel, liver, kidneys and spinal cord. **(C)** Restaging 6 month PET/CT negative. Remains on surveillance off therapy more than 3 years after treatment with a recent CEA 1.3, undetectable circulating tumor DNA and negative CT and MRI.

Radiation technique included stereotactic radiation for 69 patients to a median dose of 27 Gy (IQR 27 to 33 Gy) in a median of 3 fractions (IQR 3 to 4). Image-guided radiation therapy was administered to 84 patients to a median dose of 50 Gy (IQR 45 to 59.4 Gy) in a median of 15 fractions (IQR 10 to 28 fractions). Brachytherapy was delivered to 2 patients to a median dose of 24 Gy in a median of 4 fractions. Treatment of the primary tumor ± regional lymph nodes was administered to 47% of patients. The median cumulative GTV volume was 44.1 cc (IQR 14.1 to 117.1 cc). An example of the treatment technique and follow-up is shown in **Figure 1**.

### 3.2 Survival outcomes and patterns of recurrence

**Figure 2:**
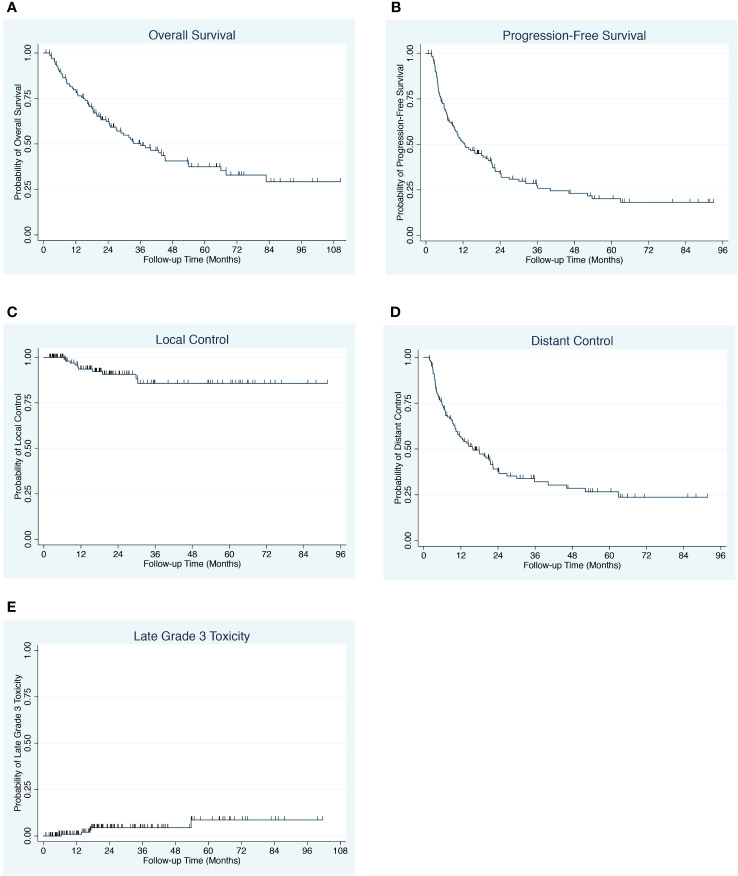
**(A)** Overall survival for patients with oligometastases. **(B)** Progression-free survival for patients with oligometastases. **(C)** Local control for patients with oligometastases. **(D)** Distant control for patients with oligometastases. **(E)** Late Grade ≥3 Toxicity for patients with oligometastases.

The median overall survival is 36.1 months with a 4-year overall survival of 41.1% (95% CI 30.6-50.5) (**Figure 2A**). The median progression-free survival was 12.5 months with a 4-year progression-free survival of 23.1% (95% CI, 15.3-31.9) (**Figure 2B**). The 4-year local control was 85.7% (95% CI, 73.9-92.5) and the 4-year distant control was 28.6% (95% CI, 19.3-38.5) (**Figures 2C, D**). On univariable and multivariable analysis, the only significant predictors of overall survival were age, performance status, favorable primary site (defined as breast, prostate and kidney) and pre-treatment serum albumin (**Table 1**, **Supplementary Table 2**). The only predictors of progression-free survival on univariable and multivariable analysis were albumin and melanoma (**Supplementary Tables 2, 3**).

Patterns of failure include 23% alive and free of disease (NED), 52% distant failure only, 9% NED but death from comorbid illness, 7% both local and distant failure, 4% NED but lost to follow-up, 4% referred to hospice before restaging, 1% local only failure, 1% alive with second primary cancer. Specific causes of comorbid death are listed in **Supplementary Table 4**. Among the 30 patients who remain alive and NED, 8 patients did not receive systemic therapy and the most common primary tumors were 9 patients with non-small cell lung cancer, 5 patients with prostate adenocarcinoma and 5 patients with breast adenocarcinoma.

### 3.3 Toxicity

Toxicities for all patients are summarized in **Table 2**. Grade 1 to 2 acute toxicities were recorded in 38% of patients. High grade acute toxicities included 1 patient with grade 3 skin toxicity and 1 patient with esophageal cancer and distant lymph node metastases who experienced grade 5 cardiac complications following esophagectomy with pathologic complete response (**Table 2**). Late grade 2 toxicities included 2 patients with radionecrosis, 1 patient with grade 2 vaginal stenosis, 1 patient with grade 2 pneumonitis, 1 patient with grade 2 erectile dysfunction and 1 patient with grade 2 urinary toxicity. Late grade ≥3 toxicities included 3 cases of grade 3 radionecrosis requiring surgery, 1 case of orthopedic screw fixation fracture and 1 case of grade 3 rectal bleeding (**Table 2**). The 4-year cumulative incidence of late grade ≥3 toxicity rate was 5% (95% CI, 2-12) (**Figure 2E**).

**Table 2:** Toxicity Following Comprehensive Involved Site Radiotherapy for Oligometastases.

Toxicity	Acute Grade 1- 2% (n)	Acute Grade ≥3 % (n)	Late Grade 1-2 % (n)	Late Grade ≥3% (n)
Gastrointestinal	13% (17)	0% (0)	2% (2)	1% (1)
Genitourinary	4% (5)	0% (0)	2% (3)	0% (0)
Neurologic	0% (0)	0% (0)	3% (4)	2% (3)
Pulmonary	2% (3)	0% (0)	2% (3)	0% (0)
Skin	16% (21)	1% (1)	1% (1)	0% (0)
Orthopedic	0% (0)	0% (0)	2% (2)	1% (1)
Fatigue	10% (13)	0% (0)	1% (1)	0% (0)
Cardiac	0% (0)	1% (1)	0% (0)	0% (0)

## 4 Discussion

The concept of curative intent radiation therapy to all areas of known metastatic disease was first proposed by Hellman and Weichselbaum in 1994 (19). By safely irradiating all areas of known disease, usually in combination with systemic therapies, a small but reproducible minority of previously incurable patients achieve long-term complete remissions (3, 10, 20). In this large single physician experience of comprehensively treating 130 patients with limited metastatic disease from 2014 to 2021, 30 patients are not only alive but without evidence of disease. While prior studies focused on the treatment of extracranial oligometastases treated with stereotactic body radiotherapy, this large real world experience included patients with oligometastases requiring treatment of the primary site, regional nodes and brain metastases (15).

In the authors’ opinion, this study better represents the entire spectrum of oligometastases in the context of patients with distant metastases referred to radiation oncology. Despite using lower biologically equivalent doses than prior studies that focused exclusively on stereotactic body radiotherapy, involved site radiation achieved durable targeted metastasis control in 86% of patients with oligometastases with an acceptable toxicity profile. Prior studies reported 63 to 87% local control at 3 to 5 year follow-up although comparisons across studies are unreliable due to heterogeneity (10, 21–24). The Duke University group reported ~90% tumor metastasis control at a median follow-up of 2 years for oligometastasis patients treated to 50 Gy in 10 fractions (16). Taken together, these data expand access to effective local oligometastasis treatment for the many clinical presentations not amenable to stereotactic body radiotherapy including those with bulky disease immediately adjacent to organs at risk. The median GTV treated in this series was 44.1 cc vs. 8.2 cc in a large multi-institutional oligometastasis database focused exclusively on stereotactic body radiotherapy (25).

While drug development continues to progress for many solid tumors, systemic therapy alone for distant metastases is generally not curative and may induce therapy-resistant genomic driver mutations (26, 27). In this series, the majority of treatment failures were the result of the development of new metastatic tumors despite advances in systemic therapy. Since isolated local failures are exceedingly rare, oligometastases may be an appropriate population to test novel therapeutics targeting either minimal residual disease or dormant micrometastases (3). Immune checkpoint inhibitors appear more effective against primary tumors and micrometastases compared to macrometastases (26). Durvulamab as consolidative treatment for stage III lung cancer following chemoradiation improves long-term overall survival and represents a potential model for this drug development strategy (28). Systematically combining comprehensive involved site radiotherapy with more effective systemic therapies represents a highly promising alternative to drug therapy alone for patients with oligometastases. Systemic therapy alone remains the standard approach for patients with >5 distant metastases with radiotherapy reserved for palliation of symptoms since subtotal metastatic ablation does not appear to alter the natural history of polymetastases (11–13).

As a single institution retrospective series of oligometastases, the patient population is inherently heterogeneous and the sample size is relatively small. The small sample size undoubtedly contributed to the inability to disprove the null hypothesis with potential predictors of progression-free survival and overall survival with radiation dose intensity, cumulative GTV volume, adjuvant systemic therapy, synchronous vs. metachronous metastases and number of metastases (**Table 1**, **Supplementary Table 2**). For the majority of these variables, there was a large numerical difference in progression-free survival but this failed to reach statistical significance. Additionally, hepatobiliary primary tumors appears to be an unfavorable primary site but did not reach statistical significance on multivariable analysis. There was no systemic therapy alone control arm so it is possible that the long-term disease-free survival and overall survival would have been similar with systemic therapy alone. Generalizability and scalability are always valid critiques of any single physician experience. On the other hand, it is well established that including opinions from a diverse group improve decision making by avoiding groupthink and the perspective of the community practitioner in academic discourse should not be ignored (29).

Although single institution and particularly single physician series are not currently in vogue, this study design has counterintuitive strengths. In contrast to large academic centers, radiation oncologists in community practice are generalists that result in practical advantages for treatment and follow-up for patients with oligometastases. High volume general radiation oncologists are facile at safely and effectively administering radiation therapy throughout the body so there is no fragmentation of care between anatomic sites (30). Since distance travelled is reduced for patients choosing care at the community setting, these suburban patients are more likely present to their local hospital rather than the urban academic medical center for acute hospitalization thus enhancing the completeness of follow-up in the context of distant metastases (31). In the specific case of specialized cancer specific hospitals, there may not be an associated emergency room and they generally will not share a common electronic medical record platform with the local primary care provider or other non-oncology specialists (32). While retrospective series are not typically associated with complete and deep record keeping, as a single physician series, these patients are extremely well known to the physician over a period of years (33). It seems likely that follow-up quality will be more complete than multi-institutional databases reflecting the experience of a large number of providers (34). Finally for this radiation oncologist with extensive experience with treatment distant metastases, selection of curative intent comprehensive metastatic ablation was informed not only by technical feasibility but also by prognosis using a validated model to supplement clinical intuition (17, 35).

In conclusion, involved site radiotherapy to all areas of known disease can safely achieve durable complete remissions in >20% of patients with oligometastases treated in the real world setting. Distant failures account for the majority of treatment failures and isolated local failures are exceedingly uncommon. Oligometastases represent a promising setting to investigate novel therapeutics targeting minimal residual disease.

## Data availability statement

The raw data supporting the conclusions of this article will be made available by the authors, without undue reservation.

## Ethics statement

The studies involving humans were approved by Good Samaritan University Hospital IRB #16-016. The studies were conducted in accordance with the local legislation and institutional requirements. Written informed consent for participation was not required from the participants or the participants’ legal guardians/next of kin in accordance with the national legislation and institutional requirements.

## Author contributions

JK: Writing – original draft, Writing – review & editing. MS: Writing – original draft, Writing – review & editing. VG: Writing – original draft, Writing – review & editing. SM: Writing – original draft, Writing – review & editing. AS: Writing – original draft, Writing – review & editing.
