# Supplementary material for: Long-term disease-free survival following comprehensive involved site radiotherapy for oligometastases
Source: Front Oncol. 2023 Dec 5;13:1267626. doi: 10.3389/fonc.2023.1267626 (PMC10739409; doi:10.3389/fonc.2023.1267626)
Supplement: Supplementary file 1 [file Table_1.docx]

**Supplemental Table 1.** Patients Stratified by Type of Systemic Therapy Received Prior to Comprehensive Radiation Therapy for Oligometastases

| **Systemic Therapy Prior to Diagnosis of Oligometastasis** | **Percent (number)** |
| --- | --- |
| None | 68% (88) |
| Chemotherapy alone | 12% (16) |
| Chemotherapy combined with biologically targeted therapy | 8% (10) |
| Hormonal therapy alone or with androgen receptor inhibitor or CDK4/6 inhibitor | 7% (9) |
| Immunotherapy or biologically targeted therapy | 5% (7) |

**Supplemental Table 2.** Univariate Predictors of Progression-Free Survival

| **Variable** | **Percent (number)** | **Median PFS (months)** | **P value** |
| --- | --- | --- | --- |
| Age, Median (range) | 71 (28 to 96) |  | 0.29 |
| <70 | 45% (58) | 14.4 |  |
| ≥70 | 55% (72) | 11.2 |  |
| Gender |  |  | 0.31 |
| Male | 54% (70) | 15.8 |  |
| Female | 46% (60) | 10.9 |  |
| ECOG performance status |  |  | 0.17 |
| 0 | 23% (30) | 35.8 |  |
| 1 | 48% (63) | 10.4 |  |
| 2 | 22% (28) | 8.8 |  |
| 3 or 4 | 7% (9) | 10.1 |  |
| Category of oligometastatic disease |  |  | 0.60 |
| Synchronous oligometastases | 40% (52) | 12.9 |  |
| Metachronous oligorecurrence or oligoprogression | 37% (48) | 20.1 |  |
| Other (induced or repeat oligorecurrence oligoprogression oligopersistence) | 23% (30) | 9.3 |  |
| Primary tumor |  |  |  |
| Lung | 35% (45) | 10.4 | 0.31 |
| Prostate | 12% (15) | 36.1 | 0.01 |
| Breast | 9% (12) | 14.4 | 0.89 |
| Colorectal | 8% (10) | 10.4 | 0.86 |
| Endometrial | 8% (10) | 7.0 | 0.45 |
| Melanoma | 5% (6) | 2.9 | 0.04 |
| Occult primary | 5% (6) | 4.8 | 0.50 |
| Hepatobiliary | 4% (5) | 7.0 | 0.04 |
| Other * | 16% (21) | 22.3 | 0.40 |
| Favorable primary tumor |  |  | 0.02 |
| Breast, prostate or kidney |  | 24.3 |  |
| All others |  | 10.1 |  |
| Metastasis location | 209 tumors |  |  |
| Bone | 31% (40) | 21.3 | 0.10 |
| Brain | 30% (39) | 7.1 | 0.15 |
| Lung | 22% (28) | 10.4 | 0.22 |
| Distant Lymph Nodes | 19% (25) | 18.5 | 0.87 |
| Liver | 10% (13) | 6.7 | 0.11 |
| Adrenal | 3% (4) | 9.3 | 0.46 |
| Albumin |  |  | 0.03 |
| ≥3.4 | 66% (86) | 19.5 |  |
| <3.4 | 28% (33) | 8.3 |  |
| Unknown | 8% (11) | 11.2 |  |
| Number of metastases treated |  |  | 0.12 |
| 0 | 7% (9) | 12.9 |  |
| 1 | 56% (72) | 12.5 |  |
| 2 to 5 | 36% (47) | 11.2 |  |
| Cumulative GTV in cm^3^, median (range) | 44.1 (0.1 to 562.6) |  | 0.15 |
| <27.7 cc | 39% (51) | 15.9 |  |
| ≥27.7 cc | 61% (79) | 12.0 |  |
| Primary tumor BED |  |  | 0.51 |
| <75 Gy | 25% (33) | 31.7 |  |
| ≥75 Gy | 22% (28) | 54.1 |  |
| Primary tumor not treated | 53% (69) | 33.4 |  |
| Average metastasis BED |  |  | 0.86 |
| <75 Gy | 61% (79) | 10.1 |  |
| 75 to 99.9 Gy | 22% (28) | 9.6 |  |
| ≥100 Gy | 18% (23) | 18.0 |  |
| Adjuvant systemic therapy |  |  | 0.76 |
| Yes | 74% (96) | 14.4 |  |
| No | 26% (34) | 7.1 |  |

**Supplemental Table 3.** Multivariable analysis for Overall Survival and Progression-Free Survival

| **Variable** | **HR for Overall Survival** | **P value** | **HR for Progression-Free Survival** | **P value** |
| --- | --- | --- | --- | --- |
| Age (continuous) | 1.05 | <0.001 | n/a |  |
| Albumin (continuous) | 0.54 | 0.005 | 0.46 | <0.001 |
| ECOG Performance Status (continuous) | 1.66 | 0.008 | n/a |  |
| Favorable Tumor Site (Breast, Prostate, Kidney vs. All Other Primary Tumors) | 2.05 | 0.037 | 1.52 | 0.14 |
| Hepatobiliary Primary | 0.36 | 0.055 | 0.54 | 0.20 |
| Melanoma | n/a | n/a | 0.23 | 0.007 |

**Supplemental Table 4.** Causes of Death Among Treated Patients Without Evidence of Active Malignancy

septic shock

urinary tract infection

renal failure on dialysis

intracranial hemorrhage

SARS-COV-2

pneumonia

post-surgical complications

sepsis and failure to thrive

renal failure

cardiac arrest while fishing

biliary obstruction

cirrhosis with encephalopathy
